# Introducing DIANA: dual-mode imaging analysis open-source simulation software

**DOI:** 10.1038/s41598-026-65869-8

**Published:** 2026-08-11

**Authors:** Oriol Sans-Planell, Shahabeddin Dayani, Nikolay Kardjilov, Ingo Manke

**Affiliations:** Helmholtz-Zentrum Berlin für Materialien und Energie GmbH, Hahn-Meitner Platz 1, 14109 Berlin, Germany

**Keywords:** Bimodal tomography, Simulation software, Bivariate histogram analysis, Reconstruction artifacts, Quantitative image quality metrics, Material segmentation, Materials science, Physics

## Abstract

The use of the complementarity of the contrast between X-rays and neutrons for computed tomography is a well-established technique. X-rays are strongly attenuated by dense, high-atomic-number materials, while neutrons are particularly sensitive to light elements, such as hydrogen or lithium. Combining the information of both modalities into a two-dimensional histogram allows for the discrimination of material phases that would otherwise not be distinguishable with a single technique alone. Here we present DIANA (Dual-mode Imaging Analysis), an open-source Python package that implements a complete dual-modality CT simulation pipeline. The current version of the software builds a voxelised phantom, creates the radiographic projections for polychromatic X-rays and monochromatic neutrons, performs controlled artifact injection and reconstruction, and finally handles the visualisation of the 2D histogram and its analysis with both shape and quality metrics. To show the capabilities of DIANA, we demonstrate the application on a simple composite phantom and a clean, high resolution study of a realistic lithium-ion cell. The architecture of the package is extensible toward other modalities, such as polarised neutrons, phase-contrast X-rays or energy-resolved neutrons, among others. The package is hosted on Github under an MIT licence.

## Introduction

Computed Tomography (CT) is a technique that reconstructs the volumetric internal structure of an object from the projection data taken around a rotation axis. The physics of the probe determines the contrast of the components of the sample. X-rays, typically in the 20–300 keV range, are mostly attenuated by photoelectric absorption, with cross-sections scaling as $Z^4$–$Z^5$ and Compton scattering, which scales linearly with the electron density of the material^1^. This interaction is ideal for visualising the contrast between high and low attenuating materials, but transparent to light materials such as hydrogen. Neutrons, on the other hand, are attenuated by nuclear cross-sections, which vary in a non-linear manner depending on the isotope and the energy of the neutrons themselves. Exceptionally large cross-sections are observed for isotopes such as hydrogen, helium-3, lithium-6, or boron-10.

Both techniques can be applied simultaneously in facilities around the world^2–4^. The resulting volume pair can then populate a 2-D histogram (also referred to as multi-modal histogram or bivariate histogram) with axis ($\mu _x$,$\mu _n$). In an ideal case, where the reconstruction is noise and artifact-free, each material populates a Gaussian cluster in the bimodal histogram plane. The spread is due to the rasterized nature of the voxel representation, and some voxels laying in the interface between materials. In that scenario, material identification can directly be done in the ($\mu _x$,$\mu _n$) space using a Gaussian mixture model, a k-means clustering or even a multi-modal Otsu segmentation^5–7^.

The quantitative analysis of bimodal histogram data has, currently, two major limitations: experimental data lacks the ground-truth that can anchor the reconstructed values of voxels in the ($\mu _x$,$\mu _n$) plane with a physical meaning. Consequently, measuring of the distance of the cluster centroids to the physical values of each material is not possible. Secondly, reliable labelled training datasets are few for AI-based reconstruction methodologies^8–10^ which have gained popularity in recent years.

We present DIANA (Dual-mode Imaging Analysis), an open-source Python framework capable of tackling both aforementioned bottlenecks currently by simulating the complete bimodal tomographic pipeline^11^. The software can create a ground truth phantom, project it using different beam geometries, probing modalities and energy spectra, inject different artifacts, reconstruct the volume, calculate the subsequent multimodal histogram, and finally analyse the result using state-of-the-art shape and quality metrics. The modularity of the framework’s architecture allows for easy scalability and inclusion of new tools and capabilities, such as polarised neutrons, phase contrast X-rays or energy-selective neutron imaging.

The potential of the framework is demonstrated with the simulation of a cylindrical lithium-ion battery with 1200 projections. DIANA introduces a label-anchored evaluation, where the centroid of each material is computed from the voxels appertaining to that material’s eroded ground-truth label mask, instead of relying on unsupervised clustering of the histogram. For a dense metallic phase (steel, copper), this label-anchored calculation reports a centroid offset of approximately 4 cm$^{-1}$ from the true attenuation values. This is a quantitative reconstruction error which is reported by the conventional Gaussian mixture model as 1 cm$^{-1}$. This discrepancy highlights the weakness of the current standard for segmentation of heterogeneous samples using clustering methods. The framework offers an alternative method, including morphological information, which results in better bimodal segmentation performance.

## State-of-the-art and related work

### Bimodal neutron—X-ray tomography

The advantages of multimodal studies using neutrons and X-rays have been clear since the early years of neutron radiography^12^. Simultaneous neutron and X-ray tomography was first introduced by LaManna et al. at NIST^13^, proving that simultaneous acquisition allowed for easier registration of volumes. Many beamlines nowadays deliver cold and thermal neutron imaging with spatial resolutions in the range of 5–200 *μ m*, with L/D values between 200 and 1000^14–16^, with many having dedicated stations for multimodal imaging^2,3^. Applications of combined neutron and X-rays are found in cultural heritage^17,18^, batteries^19,20^, fuel cells^21,22^, additive manufacturing^23,24^ and biomedical implants^25,26^, among others.

### Histogram-based multimodal analysis

The bivariate histogram as a segmentation tool has become a standard in the community, with an early formalisation by Kaestner et al.^27^, showing that Gaussian mixture models on the ($\mu _x$,$\mu _n$) plane can deliver better results than a modality-specific thresholding segmentation on cement cores. Törnquist et al.^28^ applied it for the discrimination of a bone-titanium interface and introduced quantitative figures of merit for the cluster separability. Tötzke et al^29^ employed the bivariate histogram workflow for the in-situ study of water uptake in soil for the non-invasive detection and localisation of microplastic particles in sediments. A case of operando 4D bimodal study of an all-solid state battery was performed by Vestin et al.^30^. These studies form the basis of the metrics implemented in DIANA, although none of them are adopted verbatim. The description of the ($\mu _x$,$\mu _n$) plane by a Gaussian mixture follows Kaestner et al.^27^, and is implemented here as the GMM branch of the quality metrics. The idea of condensing cluster separability into scalar figures of merit follows Törnquist et al.^28^; DIANA realises it through the Davies-Bouldin index^31^ (Eq. 24) and the pairwise material overlap $O_{ab}$ (Eq. 26), both computed on ground-truth-anchored clusters rather than on fitted components. The remaining metrics are introduced in this work: the shape metrics ($S_h$, $S_v$, $S_d$, $A_x$, $\Delta _n$), which characterise the histogram without requiring any segmentation, and the label-anchored formulations of $\varepsilon _k$, *CE*, $E_k$, $\sigma ^k$ and $O_{ab}$, in which the fitted centroids are replaced by the mean over the eroded ground-truth mask of each material. Every metric is defined in Appendix.

### Tomography simulation frameworks

Monte-Carlo transport codes such as Geant4^32^, FLUKA^33^ or MCNP^34^ provide high-fidelity transport physics but are computationally expensive for whole-volume tomographic projections, and not designed for reconstruction pipelines. These codes are cited to delimit the design space for DIANA, not as components of the framework. Attenuation is computed deterministically, by ray-tracing the Beer-Lambert integral (Eq. 2) through the voxelised phantom and summing over the energy bins of the polychromatic X-ray spectrum, while the neutron channel uses imaging-effective coefficients. Scattering, beam hardening and detector response are consequently introduced phenomenologically through the artifact model of Table 3, rather than emerging from first-principles transport. Tomography reconstruction toolkits such as ASTRA^35^ or TomoPy^36^ provide high-performance forward/back-projection operators and tools for tomography reconstruction, but do not have capabilities for phantom creation, artifact injection or histogram analysis. DIANA uses ASTRA toolbox for the GPU backend for the forward projection and the iterative reconstruction when available, and has a fallback to a simple numpy^37^ routine when not. It complements the aforementioned packages by including the phantom database, the artifact injection model and the quantitative histogram diagnostics. While it does not possess transport capabilities in the current version, DIANA offers a fast diagnostic tool to assess the quality and feasibility of multimodal experiments.

## Results

To showcase the potential of DIANA, a couple of examples are computed: a composite phantom with different materials, reconstructed using different algorithms, and a standard cylindrical lithium-ion battery. For both simulations 1200 projections were used on a phantom with 1024$^3$ voxels.

### Cross-algorithm analysis of a composite phantom

**Fig. 1:**
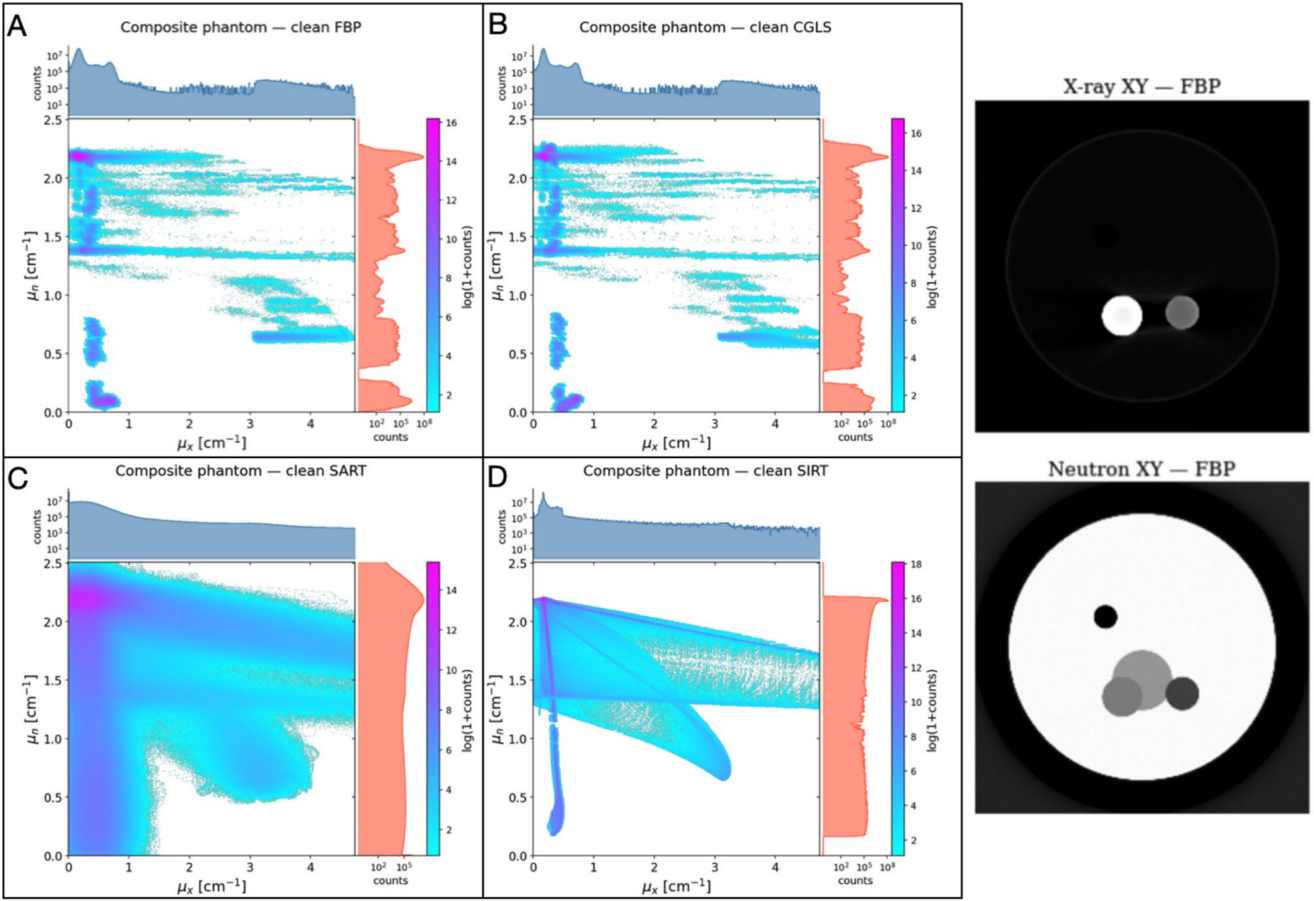
(**A**) filtered back-projection (FBP), (**B**) iterative least-squares CGLS, (**C**) SART and (**D**) SIRT. 1200 projections were computed for an identical composite phantom with size of 1024$^3$ voxels. Projections were simulated using a polychromatic X-ray with hypothetical tube voltage of 120 kVp and monochromatic thermal neutrons of 25 meV. Each bimodal histogram is accompanied by the corresponding one-dimensional histograms along the $\mu _x$ and $\mu _n$ axes, shown on a logarithmic scale to improve visualization. The metrics corresponding to the four bimodal histograms are displayed in Table 1. On the right, two central slices from the composite phantom reconstruction using the FBP, for X-rays and neutrons respectively. The complete views are shown in the supplementary material Fig. S2.

Figure 1 presents four bimodal histograms, all reconstructing the same composite sample with four different algorithms. The phantom chosen is a hollow aluminium cylinder filled with HDPE with inclusions of water, iron and titanium. All four simulations are computed on an identical phantom without introducing any artifacts nor beam hardening corrections, making the only variable the reconstruction algorithm.

We define a *usable reconstruction* as one that satisfies two conditions on the $(\mu _x,\mu _n)$ plane: each material should form a compact, localised cluster near the ground truth position for the material, and clusters of different materials must be separated. FBP and CGLS (Fig. 1A,B) satisfy both conditions, as distinct clusters are formed (albeit streaks in the horizontal axis) with locations compatible with the ground truth attenuation values. SIRT (Fig. 1D) fails on the first condition, as the two triangular structures span most of the $(\mu _x,\mu _n)$ domain. SART (Fig. 1C) performs even worse than SIRT: the diffuse, nearly uniform blob suggests that the algorithm converged to an over-smoothed solution, suppressing the contrast differences between the different materials.

The values for $S_h$ (Eq. 8) and $S_v$ (Eq. 9) reported in Table 1 are very elevated for all algorithms, especially for an artifact-free simulation. These values are an indication on the nature of the composite sample, which is the high-contrast materials present in it. As very high-Z materials (Fe, Ti) are embedded in very light material matrix (HDPE, H$_2$O), even in absence of misalignment or other artifacts, the back-projection operator will tend to redistribute attenuation coefficients from the metal into the matrix, generating the streaks and introducing the bias in $S_h$ and $S_v$. The diagonal smear $S_d$ (Eq. 12) is close to zero for FBP, CGLS and SIRT, indicating no correlated drift of the two modalities, which is consistent with the neutron channel being monochromatic, so the polychromatic X-ray bias cannot correlate with a neutron bias. The positive SART values (0.63) shows an over-smoothed solution, since the voxel values migrate toward population-averaged ($\mu _x$,$\mu _n$) pairs, making both axes artificially correlated. This highlights the importance of the multiple metrics, as Davies-Bouldin index *DB* (Eq. 24) and per-material elongation $E_k$ (Eq. 19) properly distinguish the streak-full yet cluster preserving FBP and CGLS, contrary to the absence of proper clusters in SIRT and SART.

Metrics reported in Table 1 show a complex evaluation problem: the mean centroid error *CE* (Eq. 21) is showing better results for SIRT (1.08 cm$^{-1}$) and SART (1.11 cm$^{-1}$), lower than FBP and CGLS (both at 1.30 cm$^{-1}$). On the other hand, the DB index indicates that the clusters generated by FBP and CGLS (0.20 and 0.21 respectively) have better separability than those made by SIRT and SART. From the perspective of the material cluster elongation $E_k$, the algebraic methods come on top, with values much closer to one, with respect to FBP and CGLS, but with the tradeoff of moving every voxel towards population-averaged values, which eliminates the cluster structure. One anomaly shown by the metrics is the Per-material elongation of aluminium. Since the aluminium is present as a thin hollow shell of the phantom, the interior mask after the erosion is dominated by voxels close to two interfaces (air and HDPE). This effect is tackled differently by each algorithm, as the cluster shape is dominated by partial-volume and edge behavior, rather than streaking. With a reduced number of voxels, the sample covariance alters the values of $E_k$.

This exercise has been made with a number of iterations of SART and SIRT of 50, and 30 for CGLS. These numbers were chosen as exemplary for the comparison of the algorithms. A more in-depth comparison between the iterative algorithms, with different number of iterations, is shown in the supplementary materials, in particular in Fig. S1 and Table S1.

**Table 1:** Ground truth-based metrics for the cross-algorithm comparison, computed on the composite phantom ($1024^3$, 1200 projections, clean polychromatic 120 kVp x-rays and thermal neutrons at 25 meV, no artifacts injected).

Metric	FBP	SIRT	SART	CGLS
Global quality scalars				
CE (cm$^{-1}$)	1.30	1.08	1.11	1.30
DB	0.20	0.52	0.64	0.21
Shape metrics				
$S_h$	134.5	155.7	241.1	194.3
$S_v$	138.7	161.0	232.6	201.8
$S_d$	−0.01	0.00	0.63	−0.01
Per-material centroid error $\varepsilon _k$ (cm$^{-1}$)				
$\varepsilon _k(\textrm{Al})$	0.34	0.43	0.43	0.34
$\varepsilon _k(\textrm{HDPE})$	0.00	0.04	0.07	0.00
$\varepsilon _k(\mathrm {H_2O})$	0.08	0.36	0.42	0.08
$\varepsilon _k(\textrm{Fe})$	4.04	3.09	3.12	4.06
$\varepsilon _k(\textrm{Ti})$	2.02	1.48	1.49	2.03
Per-material elongation $E_k$ (label-anchored)				
$E_k(\textrm{Al})$	4.5	5.3	1.4	2.9
$E_k(\textrm{HDPE})$	4.4	2.0	2.5	2.6
$E_k(\mathrm {H_2O})$	30.3	7.0	6.4	19.6
$E_k(\textrm{Fe})$	122.6	3.1	3.6	95.3
$E_k(\textrm{Ti})$	29.3	1.5	2.0	16.2

All metric values are quoted to two decimal places, with label-anchored metrics, $S_h$ and $S_v$ shown with one decimal place. The lower block shows per-material elongation $E_k$ (label-anchored), where a value near unity indicates a compact, isotropic cluster, and large values indicate streak-distributed reconstructions. The two algorithms with the lowest mean centroid error (SIRT, SART) are precisely those that appear unreliable on a visual inspection at the bimodal histogram, and their CE-rank advantage arises from voxels reconstructing toward population-averaged *μ* values rather than toward each material’s true centroid.

From this small experiment, it is shown that no single metric of $\{CE, DB, S_d, E_k\}$ can faithfully quantify the goodness of the histogram. Depending on the sample, materials, reconstruction algorithm and artifacts, every metric can show a different aspect. The combination of all of them, along with the visual evaluation of the histogram can report a complete story. For the simulated case of the composite phantom, at this resolution, FBP and CGLS seem to perform better than SIRT and SART, even though the metallic phases, such as Ti and Fe are reconstructed as streaks. The streaks can be partially mitigated using beam hardening corrections, as part of the effect comes from the polychromatic hardening of the X-rays, in addition to the metal-induced streaks that persist even for monochromatic projections.

This example highlights the potential of DIANA to deliver a deep analysis, capable of optimising the configuration for an experiment to maximise the quality of the results.

### Lithium-ion battery

**Fig. 2:**
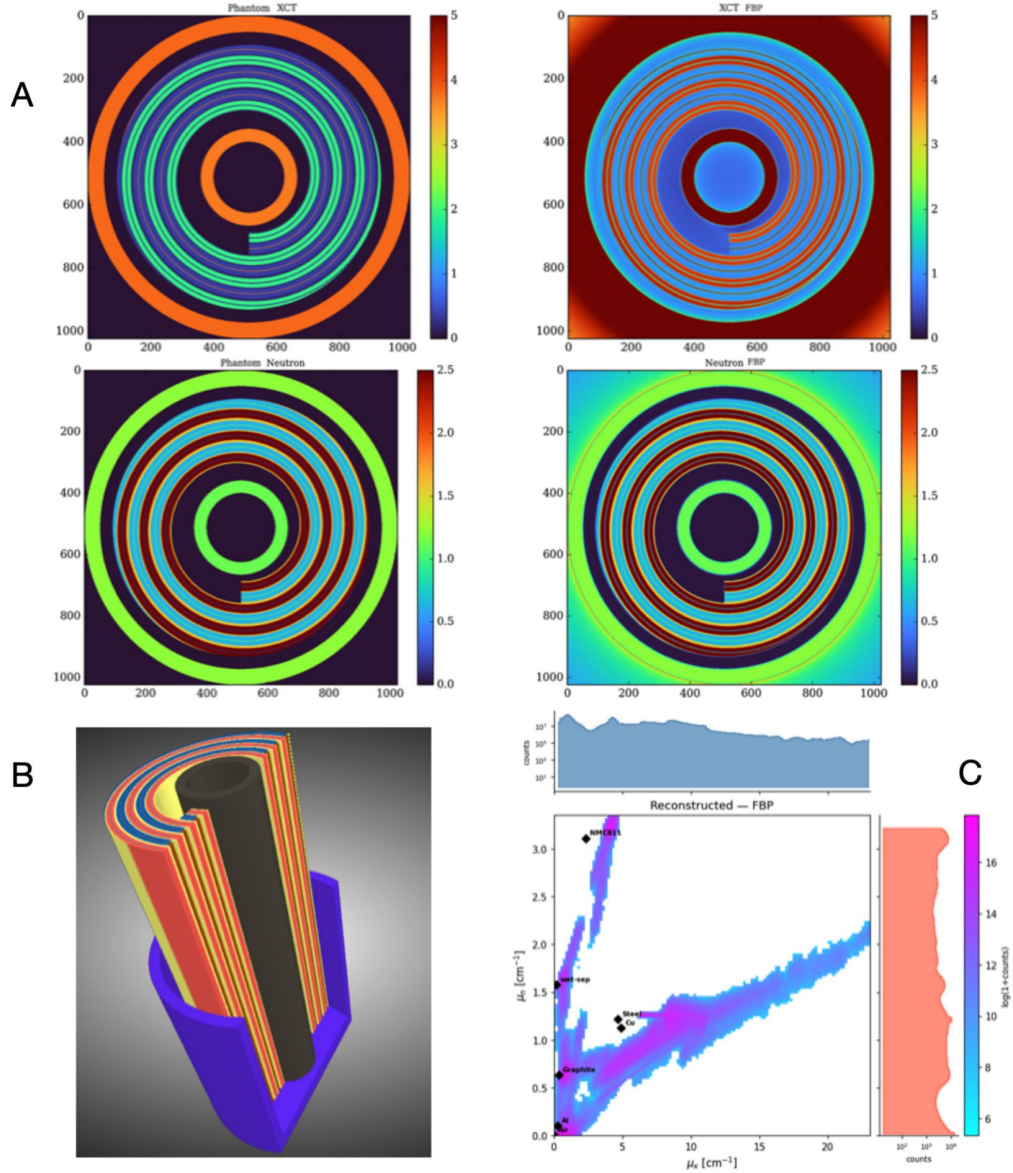
(**A**) Ground truth visualisation of the phantom with neutrons and X-rays with the respective FBP reconstruction. (**B**) 3D model showing the ground truth phantom and a highlight of the different materials. (**C**) 2-D histogram of the registered reconstructed volumes. The reconstruction has been done without including any artifacts. The bimodal histogram shows the true location of the materials, and highlights how certain material clusters are moved from the true positions due to the effects of the reconstruction.

For a realistic case, we simulated a cylindrical lithium-ion cell phantom, with a steel can and end disks, copper current collector, NMC-811 cathode, graphite anode and a separator. Figure 2A shows three different slices from the ground truth volume, while (B) shows a 3D visualisation of the complete phantom. No artifacts were added in the simulations. Analysis was done with both the GMM and the labelled-anchored metrics in order to compare their performances. The GMM was computed by initialising a 12-component mixture using the ground-truth material locations and matching to greedy nearest-neighbour assignment. The label-anchored metrics calculate the centroids from the eroded ground-truth label mask of each material, without clustering first. Results are shown in Table 2. Both metrics agree on the low-Z materials, with relatively small *CE*, but differ on the high-Z. The GMM reports $\varepsilon _{Cu}=0.52 \,\textrm{cm}^{-1}$ and $\varepsilon _{steel}=0.72 \,\textrm{cm}^{-1}$ (Eq. 20), while the label-anchored shows $\varepsilon _{Cu}=3.85 \,\textrm{cm}^{-1}$ and $\varepsilon _{steel}=4.00 \,\textrm{cm}^{-1}$. The ground truth coordinates for Cu and Steel are (4.92, 1.12) cm$^{-1}$ and (4.70, 1.21) cm$^{-1}$ respectively, tabulated at 80 keV, with the reconstructed centroids at (8.75, 1.10) cm$^{-1}$ and (8.69, 1.19) cm$^{-1}$. Values for $\mu _x$ are therefore biased by roughly 4 cm$^{-1}$, while for $\mu _n$ they are recovered correctly. Part of this displacement reflects the energy convention: the ground truth is anchored at the tabulated 80 keV value, whereas a polychromatic reconstruction treated as monochromatic converges towards the effective energy of the beam, which for the 120 kVp spectrum used here lies near 67 keV, where $\mu _x(\textrm{Cu})$ is approximately 9 cm$^{-1}$. The label-anchored metric measures the total displacement between the two, which the Gaussian mixture model under-reports by a factor of seven. Values for *CE* between the GMM and the label-anchored metric disagree by a factor of 2.4, while in case of DB metric the difference is a factor of 11 (0.34 vs 4.24).

The two strategies differ strongly on the cluster morphology. The GMM reports a point-like copper cluster ($\sigma _x(\textrm{Cu})$ = 0.00 cm$^{-1}$, Eq. 22) because it is fitting a compact component near the displaced voxel population, while the label-anchored calculation shows the correct dispersion of the copper voxels along $\mu _x$. Another sharp discrepancy is found in the pairwise overlap $O(\textrm{Steel,Cu})$ (Eq. 26), where the GMM shows complete overlap, while the label-anchored reports a value of 0.48, meaning that roughly half of the metal voxels are assigned to the wrong phase, even when the centroids are anchored to the eroded ground-truth masks.

The Gaussian Mixture Model under-reports on the metrics compared to the label-anchored approach. This is due to high-contrast phases present in the sample, whose voxels get reconstructed far away from their true values in the histogram, and get associated, wrongly, with other materials. The GMM is more sensitive to these effects than the labelled-anchored approach.

**Table 2:** Side-by-side metric output on a clean $1024^3$, 1200-projection FBP reconstruction of the lithium-ion cell.

Quantity	GMM	Label-anchored
Global quality scalars		
CE (cm$^{-1}$)	0.82	2.01
DB	0.37	4.24
Per-material centroid error $\varepsilon _k$ (cm$^{-1}$)		
$\varepsilon (\textrm{Cu})$	0.52	3.85
$\varepsilon (\textrm{Steel})$	0.72	4.00
$\varepsilon (\textrm{NMC811})$	1.36	1.95
$\varepsilon (\textrm{Al})$	0.83	0.84
$\varepsilon (\mathrm {Sep{+}Etl})$	0.83	0.72
$\varepsilon (\textrm{Graphite})$	0.67	0.70
Per-material cluster spread		
$\sigma _x(\textrm{Cu})$ (cm$^{-1}$)	0.00	0.59
Pairwise overlap		
$O(\textrm{Steel,Cu})$	1.00	0.48

The metrics agree on low-contrast phases (Al, Separator+Electrolyte, Graphite) and disagree on the dense metallic phases (Cu, Steel) and on the global summary scalars CE and DB.

## Discussion

DIANA offers a GPU-capable, open-source framework for studying single- and multimodal samples measured with X-rays and neutrons. Combining these modalities enhances the segmentation and identification of different material phases, while accurate sample simulations can provide additional information that improves experimental insight and outcomes.

The software is built using a modular architecture, designed from the beginning to allow a scalable development by adding more capabilities, such as additional imaging modalities, new geometries, different physics engines or alternative analysis techniques. Three extensions are planned for future releases: energy-selective neutron imaging by integrating the NCrystal package^38^, which would enable energy-resolved imaging and to resolve crystallographic information; polarised neutron imaging^39^, by generalising the scalar $\mu _n$ to a *2 × 2* line-integrated magnetic field; and propagation- or grating-based phase contrast X-rays, by adding a refractive index *δ* to $\mu _x$ which would resolve the issue of overlapping low-Z clusters in the bimodal space. Two further improvements that will be developed alongside the ones listed are a CAD and segmented volume import functionalities, to create realistic phantoms from existing stl or labelled models, and the integration of a McStas-like transport code for the neutron spectrum and the scatter components, which would substitute the current analytical version, making it more similar to experiments.

As the code is delivered, there are two immediately available resources to the community: an artifact fingerprint library that can let instrument scientists and researchers to identify dominant artifacts in real bimodal data; and a synthetic training data pipeline for supervised reconstruction and segmentation algorithms that large scale facilities increasingly adopt. Each simulation run provides a labelled triple volume, composed of X-rays, neutrons and ground truth, which is exactly the input format necessary for supervised segmentation or reconstruction networks^40^. Since every volume brings a labelled ground truth phantom, the previously unmeasurable centroid distance becomes quantifiable as a figure of merit. Volumes can be generated with any combination and intensity of artifacts, reconstruction techniques or materials, allowing for thousands of volumes to be prepared for a single sample. This case can be very positive for the battery community, where access to large-scale facilities is expensive and scarce.

The objective of DIANA is threefold, on increasing time scales. In the immediate term it supplies a controlled ground truth: a labelled (X-ray, neutron) volume pair in which every displacement of a histogram cluster from its true position can be attributed to a known cause, which is not achievable with experimental data alone. In the medium term, the artifact fingerprint library is intended to be used in reverse: by matching the shape metrics of an experimental bimodal histogram against the simulated fingerprints, the dominant artifact in a real dataset can be identified and the appropriate correction (beam hardening polynomial, ring correction, registration, ...) selected on a quantitative basis instead of by visual inspection. In the long term, the labelled triples generated by the framework are intended as training data for supervised reconstruction, segmentation and analysis networks. Delivering the second and third of these objectives requires DIANA to be benchmarked against data from a simultaneous neutron and X-ray beamline, which is the immediate next step of this work.

## Methods

**Fig. 3:**
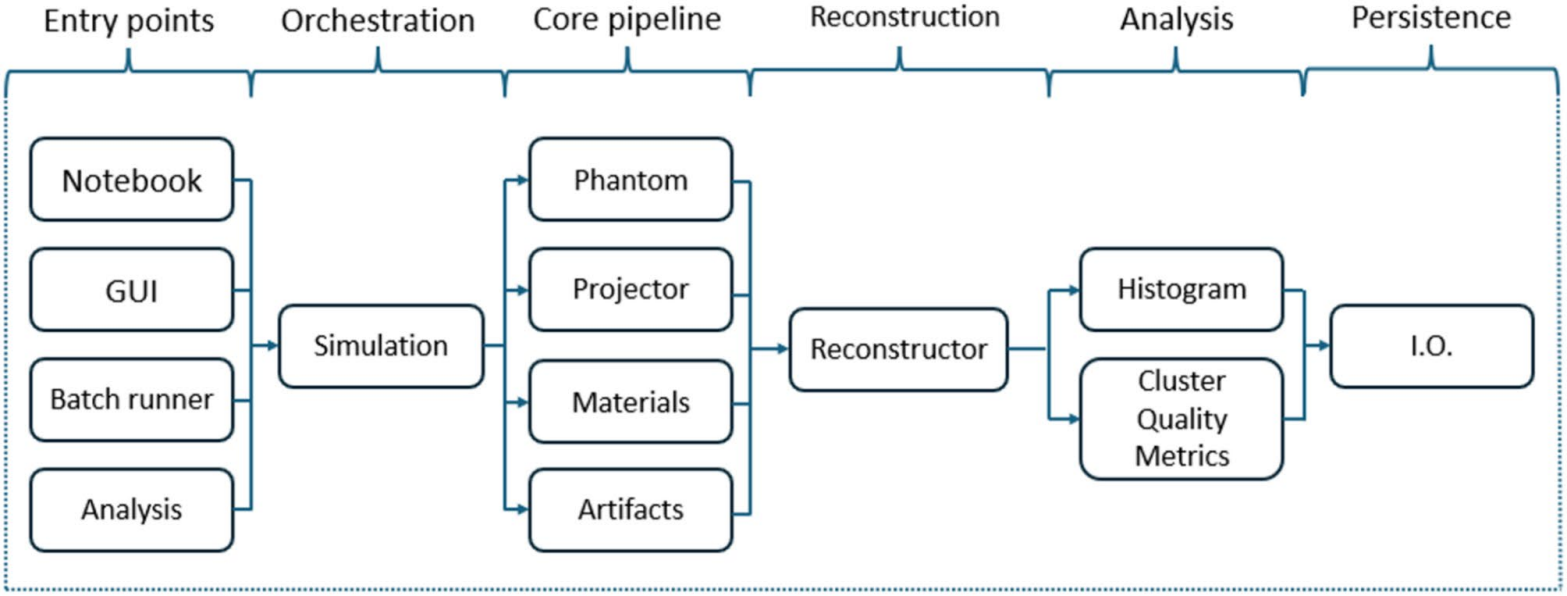
The complete architecture of DIANA. The pipeline is organised in six layers: Entry points, providing user input and interaction; the orchestration, which coordinates the core pipeline (Phantom, Projector, Materials and Artifacts modules); the reconstruction and analysis which handle the shape and quality metrics calculations; and finally the I/O module, which is in charge of visualisation and storage of the results.

DIANA is programmed in Python, with a modular architecture that allows easy scalability, flexibility for new module implementation, and follows a straightforward pipeline. The software diagram is shown in Fig. 3. The framework offers different user interaction possibilities. The API of the package enables programmatic use of the software as shown in sample notebooks. In addition, a GUI for phantom generation and data analysis is also available in this software package. There are also tools for batch processing and post-processing data analysis. The different modules are independent from each other, and they can be called or modified without risk of altering the overall work of the framework.

### Materials database and X-ray spectrum

The material database provides a list of materials, parametrised by their mass density, three neutron attenuation coefficients, corresponding to absorption, coherent and incoherent scattering, and a 13-point X-ray linear attenuation table in the range 20–300 keV. The tabulated data is extracted from the NIST XCOM database^41^. The neutron values employ imaging-effective coefficients, accounting for the fact that a transmission detector with a finite solid angle coverage captures a non-negligible fraction of forward-scattered neutrons^42^. Users can extend the database by including more *Material* dataclass instances.

The polychromatic X-ray spectrum is generated in three steps, starting with the use of the thick-target bremsstrahlung approximation for CT simulation^43–45^:

1 $$\begin{aligned} S(E)\propto Z_WE(kVp-E), \end{aligned}$$

where *S*(*E*) is the unfiltered bremsstrahlung spectrum, E is the photon energy in keV, $Z_W$ the atomic number of the anode target (set at 74 for tungsten) and kVp is the maximum kinetic energy (in keV) that an accelerated electron carries when hitting the anode, thus setting the higher cut-off energy of the spectrum.

The spectrum is then filtered, according to the following equation:

2 $$\begin{aligned} T(E)=exp[-\mu _{Al}(E) \,t_{Al}-\mu _{Cu}(E) \,t_{Cu}]. \end{aligned}$$

*T*(*E*) is the transmission through a stack of pre-filter foils, in the range [0, 1]; $\mu _{Al}(E)$ and $\mu _{Cu}(E)$ are the linear attenuation coefficients, expressed in cm$^{-1}$, for aluminium and copper and photon energy E, respectively; and $t_{Al}$ and $t_{Cu}$ are their thicknesses, in *cm*. Equation (2) is a direct application of the Lambert-Beer law of attenuation.

The weights of the different channels are then normalised, following:

3 $$\begin{aligned} W(E)=\frac{S(E) T(E)}{\sum _{E^\prime } S(E^\prime )T(E^\prime )}, \end{aligned}$$

where *W*(*E*) are the normalised weights, considering $\sum _E W(E)=1$; the numerator component *S*(*E*)*T*(*E*) is the filtered photon flux at a certain energy *E*; and the denominator component is the sum over all energy bins.

The resulting sinogram is calculated by integrating the normalised spectra across all the material of the sample, depending on the geometry:

4 $$\begin{aligned} I(r_{det})=\sum _E W(E)exp[-\int \mu _x(E,r)dr], \end{aligned}$$

where $I(r_{det})$ defines the intensity over a certain ray $r_{det}$. In case of the monochromatic spectra, Eq. (4) is simplified to a single energy value. This simplified application is used for neutrons, and can be expanded for energy-selective neutron imaging, as argued in the Discussion Section.

### Phantom generation

The *PhantomBuilder* class offers a basic functionality for phantom creation which allows for the making of general-shape samples within a defined space with a user-defined Width x Depth x Height voxel number. Each material is then assigned a material index and the consequent attenuation coefficient are derived afterwards. The material parameters of both phantoms, with their provenance, are listed in Supplementary Table S2.

### Projection and artifacts

Two separated projection modalities are implemented in DIANA: the main one using ASTRA toolbox and its CUDA-based parallel-beam functions, which individually performs the projection of the multiple X-ray energies and sums them at the sinogram (Eq. 4); and a numpy-based fallback, for systems which are either not GPU-capable or do not have ASTRA toolbox installed. The projection of the neutron data follows the same scheme, including the calculation of the three components of the beam. There is also the implementation of the cone-beam geometry, appropriate for laboratory-CT simulations or realistic low-L/D neutron imaging. The cone-beam geometry is done using ASTRA’s fan-projection function, which simulates a 2D fan beam, which is then iterated over every slice. This gives a sufficiently good approximation for the cone-beam projection geometry, while remaining computationally inexpensive.

**Table 3:** Artifact model implemented by DIANA.

Domain	Artifact	Key parameters	Bimodal histogram effect
Sinogram	Poisson photon/neutron noise	$I^{0}_{xrays}$, $I^{0}_{neutrons}$	Isotropic cluster broadening
Sinogram	X-ray beam hardening	Polychromatic spectrum	Horizontal smearing
Sinogram	Neutron scatter	$scatter\_fraction$, $scatter\_sigma\_pixels$	Upward shift along $\mu _n$ axis
Sinogram	X-ray scatter	$xray\_scatter\_fraction$, $xray\_Scatter\_sigma$	Diagonal smear, loss of contrast
Sinogram	Detector PSF	$psf\_sigma\_xray$, $psf\_sigma\_neutron$	Partial-volume line thickening
Sinogram	Ring artifacts (X-ray)	$n\_bad\_column$, $ring\_amplitude$	Vert. stripes at isolated *μ* values
Sinogram	Ring artifacts (neutron)	$n\_bad\_column$, $ring\_amplitude$	Hor. stripes at isolated *μ* values
Volume	Rigid-body misalignment	$translation\_voxels$, $rotation\_deg$	Horizontal streaks
Volume	Salt-and-pepper voxel noise	$salt\_pepper\_fraction$	Diffuse background

Each row represents one possible artifact to be added to the simulation through the *ArtifactConfig* class. The fourth column shows the ideal effect to be expected for each artifact.

Photon and neutron counting noise are considered Poisson processes on the intensity of the sinogram before the *-log* transformation, with a mean per pixel per projection equal to $I_0\cdot I(r_{det})$, where $I(r_{det})$ is the polychromatic transmitted intensity of Eq. (4). Beam hardening appears as a consequence of the polychromatic projection over different materials and it is optionally corrected using a polynomial spline fit of order 2–4. The neutron scatter is modelled as a Gaussian blurring with the intensity map derived from the scatter fraction. X-ray scatter has a similar structure as the neutron scatter component. Ring artifacts are additive offsets on a small subset of sinogram columns before the reconstruction, and can be corrected using a column-normalisation algorithm^46^. Rigid-body misalignment is an affine transform (translation plus Euler rotation) of one of the volumes with respect to the other.

### Reconstruction

Nine reconstruction algorithms are implemented in DIANA: Filtered Back-Projection, implemented with selectable filters (Ram-Lak, Shepp-Logan, Hann, Cosine and Hamming); Gridrec^47^, from Tomopy; SIRT^35,48^; SART^49^; CGLS^50^; EM^51^; ordered-subset SART (OS-SART)^52^; total-variation minimisation^53^; and Nesterov-accelerated SIRT^54^. All algorithms rest on ASTRA’s GPU when available, and rely on scikit-image’s Radon transform when not.

### Bimodal histogram, shape and quality metrics

The bimodal histogram is calculated by flattening the $(\mu _x,\mu _n)$ voxel pairs into a user-defined grid. Two kinds of metrics are then used to evaluate the histogram: shape metrics which define the histogram independently from any segmentation, and quality metrics, which quantify the alterations on the histogram with respect to a ground-truth labelled volume pair.

Shape metrics are the following: horizontal streak score ($S_h$), vertical streak score ($S_v$), diagonal smear score ($S_d$), marginal asymmetry of $\mu _x$ ($A_x$) and the marginal shift of $\mu _n$ ($\Delta _n$). The quality metrics operate on two levels: first a Gaussian Mixture Model, as defined in literature for the analysis of bimodal histograms, and secondly what was called a labelled-anchored modality, to compute the per-material centroids from the mean of $(\mu _x,\mu _n)$ over the eroded ground-truth labelled mask of each material (hence the name). The metrics calculated by the quality metrics are: Euclidean distance from centroid to ground truth position $\varepsilon _k$, mean centroid error *CE*, Davies-Bouldin index *DB*, per-material cluster elongation $E_k$, cluster spread $\sigma _x^{k}$ and $\sigma _n^{k}$ and the pairwise material overlap $O_{ab}$.

All metrics are described in detail in Appendix.

## Supplementary Information

Supplementary Information.

## Data Availability

The datasets used and/or analysed during the current study are available from the corresponding author on reasonable request.

## Appendix

### Mathematical description of the metrics

The calculation of the bimodal histogram is computed by flattening the two volumes and building the plot in a user-defined bin grid number.

Some necessary notation before defining the metrics used to evaluate the quality of the histogram need to be defined: A bimodal histogram generates, for each of the V voxels of the reconstructed volume, a pair of attenuation coefficients ($\mu _x$,$\mu _n$), expressed in cm$^{-1}$. For this section, indices $v\in \{1,...,V\}$ define the voxels.

The histogram is computed on a uniform grid of *B* bins, where $b_x\in \{1,...,B_x\}$ and $b_n\in \{1,...,B_n\}$ iterate over each bin, with centres $x_i$ for $i\in (1,...,B_x)$ and $y_j$ for $j\in (1,...,B_n)$ for X-rays and neutrons, respectively.

The unnormalised histogram count in a certain cell (*i*, *j*) is written as $H_{ij}$, with the normalised version being $p_{ij}=H_{ij}/\sum _{ij}H_{ij}$, so that $\sum _{ij}H_{ij}=1$. The corresponding marginals are:

5 $$\begin{aligned} p_x(x_i)=\sum _jp_{ij},\quad p_n(y_j)=\sum _ip_{ij}. \end{aligned}$$

The phantom has a ground-truth label volume $L_v\in {1,...,M}$ which assigns to each voxel a material *M*, with a known material attenuation pair:

6 $$\begin{aligned} C_{k}^{GT}=(\mu _x^k,\mu _n^k)\in \mathbb {R}^2,\quad k\in {1,...,M}. \end{aligned}$$

Two different metrics are used for the analysis of the histogram: Shape metrics and quality metrics. The former rely only on the histogram itself, thus being applicable to both simulated and real data. The latter use the ground-truth positions and the label volume, therefore are only computable on the simulation.

#### Shape metrics

Shape metrics are independent from the ground truth and evaluate solely the histogram data.

##### Horizontal streak metric

When a volume is misaligned, either translated or rotated with respect to the other, voxels from X-rays are wrongly paired with the neutron counterpart. With the convention that the frame of reference is the X-ray volume, this misalignment will generate horizontal streaks in the ($\mu _x$,$\mu _n$) plane. For every row *j* of the normalised histogram, we define the row variance as:

7 $$\begin{aligned} V_{row}(j)=(1/B_x)\sum _i(p_{ij}-\overline{p}_j)^2,\text { with }\overline{p}_j=(1/B_x)\sum _ip_{ij}. \end{aligned}$$

And the analogous column variance $V_{col}(i)$. The horizontal streak metric $S_h$ is the maximum row variance normalised by the mean column variance, as follows:

8 $$\begin{aligned} S_h=\frac{max_jV_{row}(j)}{(1/B_x)\sum _iV_{col}(i)}. \end{aligned}$$

When the histogram is approximately isotropic in the ($\mu _x$,$\mu _n$), then $S_h\approx 1$. $S_h\gg 1$ if one or more rows have a much higher variance than other columns, which is the geometry of a horizontal streak.

##### Vertical streak metric

This metric works as an indicator of ring artifacts. Detector columns with a biased gain caused by burned or defective pixels create straight lines on the sinogram that translate into rings on the volume. These rings appear on the 2-D histogram as straight lines, that can be either vertical or horizontal, depending on the modality: a ring in the X-ray volume will produce a vertical streak at the biased $\mu _x$ value, as all the pairs ($\mu _x$,$\mu _n$) will have a wrong X-ray attenuation coefficient, with correct neutron ones. The system works in the same way for neutrons, producing horizontal streaks. The metric is calculated as the counterpart of $S_h$ in Eq. (8):

9 $$\begin{aligned} S_v=\frac{max_iV_{col}(i)}{(1/B_n)\sum _jV_{row}(j)}. \end{aligned}$$

In absence of misalignment, the shape-metric ratio $S_v/S_h$ identifies the dominant ring channel. In case of misalignment being present in this application, it is expected that $S_h\gg S_v$, as the streaks would combine for both artifacts. A clean reference should have $S_v\approx S_h\approx 1$.

##### Diagonal smear metric

In order to detect X-ray beam hardening or other artifacts that can create a bias on both modalities in correlated directions, we evaluate the smear over the diagonal which is quantified by Pearson’s correlation between two conditional-mean curves. The marginal-mass-weighted mean of $\mu _x$ per neutron bin is defined as:

10 $$\begin{aligned} \overline{X}(y_j)=\sum _ip_{ij}x_{i}. \end{aligned}$$

The $$\mu _n$$ counterpart:

11 $$\begin{aligned} \overline{Y}(x_i)=\sum ^{B_n}_jp_{ij}y_{j}. \end{aligned}$$

The two curves have different lengths, $B_n$ and $B_x$ respectively, so $\overline{Y}$ is linearly resampled onto the parametric grid *s∈ [0,1]* of length $B_n$. The diagonal smear metric is calculated then from the Pearson correlation:

12 $$\begin{aligned} S_d=\rho (\overline{X},\overline{Y})\in [-1,1]. \end{aligned}$$

$S_d$ would be close to 0 for an artifact-free reconstruction, as cluster positions would be fixed on certain ($\mu _x$,$\mu _n$) values and not be smeared by different bias. A positive $S_d$ would indicate a positively correlated drift, indicating polychromatic beam hardening. Negative $S_d$ values would show an anti-correlated bias, which could indicate high neutron scattering with a monochromatic X-ray spectrum. In the code implementation, if either $\overline{X}$ or $\overline{Y}$ have zero variance, the algorithm returns $S_d = 0.00$.

##### X-ray marginal asymmetry

It highlights the radial-direction asymmetry caused by the polychromatic beam hardening reconstructions where the centre of a homogeneous region is assigned a lower $\mu _x$ value compared to the outer section. To calculate the asymmetry, we use the marginal $p_x(x)$, compute the bin-weighted mean and the median:

13 $$\begin{aligned} \hat{\mu _x}=\sum _ip_x(x_i)\cdot x_i,\quad \hat{m_x}=x_{i^*}\text { with }i^*=min\{i:\sum _{k\le i}p_x(x_k)\ge 1/2\}. \end{aligned}$$

The asymmetry is then calculated as the standardised mean-median offset:

14 $$\begin{aligned} A_x=(\hat{\mu _x}-\hat{m_x})/\sigma [\mu _x], \end{aligned}$$

where $\sigma [\mu _x]$ is the standard deviation of the voxel $\mu _x$ values. For a symmetric distribution (an ideal, clean reference) it would be expected that mean *≈* median and that $A_x\approx 0$. Beam hardening will pull the voxel values in dense regions to lower $\mu _x$, while the peripheral voxels will remain with the correct values. This will generate a long tail toward the higher $\mu _x$ and make $A_x> 0$. This formulation is less sensitive to alterations due to salt-and-pepper artifacts, which would populate the tails of the histogram.

##### Neutron marginal shift

Neutron scattering component alters the values of the pixels by increasing the measured transmission, which reduces the values of the line integral after applying the *-log* operation during reconstruction, that in turn increases the values of $\mu _n$. This bias can be approximated to a uniform impact across materials, shown as a global shift of the neutron marginal. To evaluate it, a clean reference $p_{ij}^{ref}$ is used, along the current histogram $p_{ij}$. The marginal shift is defined as the difference between voxel mean $\mu _n$ values:

15 $$\begin{aligned} \Delta _n=\langle \mu _n\rangle _{current}-\langle \mu _n\rangle _{ref}. \end{aligned}$$

By definition, $\Delta _n\approx 0$ for clean data; $\Delta _n> 0$ would indicate the presence of a scatter component, while $\Delta _n< 0$ would warn of a normalisation error, since it is unlikely to have a scatter component that is lower with respect to a clear volume. This is the only shape metric that requires a reference.

#### Quality metrics

Contrary to the previously defined shape metrics, quality metrics require information of the ground truth in order to evaluate the goodness of the histogram and its components.

##### Per-material cluster elongation

Characterises the anisotropic elongation of an individual material’s cluster in the $(\mu _x,\mu _n)$ plane. It requires ground-truth knowledge and it returns a vector $\{E_0,E_1,...,E_M\}$ describing the elongation *E* of each material. To calculate it, it is necessary to first define the following eroded interior mask:

16 $$\begin{aligned} V_{k^{int}}=erode_r({v:L_v=k}), \end{aligned}$$

where $erode_r$ applies an erosion mask on the border of the cluster with radius *r*. The label-anchored centroid for a material *k* is the mean of $(\mu _x,\mu _n)$ inside the mask, thus:

17 $$\begin{aligned} \hat{\mu }_k=(1/|V_{k^{int}}|)\sum _{v\in V_{k^{int}}}(\mu _x^{(v)},\mu _n^{(v)}). \end{aligned}$$

The erosion prevents the boundary ambiguity meaning that, in case of a voxel at the intersection between materials *k* and *k'*, the voxel would be eliminated by the mask, preserving the location of the centroid.

Knowing $V_{k^{int}}$ for each *k*, and defining $p_v=(\mu _x^{(v)},\mu _n^{(v)})$, with $\hat{\mu }_k$ as the label-anchored centroid of the cluster, the covariance matrix is written as follows:

18 $$\begin{aligned} \hat{S}_k=(1/|V_{{k^{int}}}|)\sum _{v\in V_{k^{int}}}(p_v-\hat{\mu _k})(p_v-\hat{\mu _k})^T. \end{aligned}$$

From here, let $\lambda _1(k)\ge \lambda _2(k) > 0$ be its sorted eigenvalues. The material elongation vector is defined as:

19 $$\begin{aligned} E_k=\sqrt{[\lambda _1(k)/\lambda _2(k)]}\ge 1, \end{aligned}$$

where $E_k=1$ would represent a circular cluster, $E_k\gg 1$ a strongly elongated one.

##### Per-material centroid error and mean centroid error

For each material whose set is not empty, the per-material centroid error is defined as:

20 $$\begin{aligned} \varepsilon _k=||\hat{\mu }_k-c_k^{GT}||=\sqrt{(\hat{\mu }_x^{(k)}-\mu _x^{(k)})^2+(\hat{\mu }_n^{(k)}-\mu _n^{(k)})^2}. \end{aligned}$$

The mean centroid error *CE* is then defined across all *M'* materials (excluding air):

21 $$\begin{aligned} CE=(1/M')\sum _k \varepsilon _k. \end{aligned}$$

Both *CE* and $\varepsilon _k$ have units of cm$^{-1}$, as they are calculated directly from the values of the voxels in the bimodal histogram. These two quantities reflect the goodness of the reconstruction with respect to the ground truth, identifying when one or more materials get clustered away from their real coordinates, be it due to an effect of the reconstruction algorithm or due to artifacts.

##### Per-material cluster spread

It calculates the standard deviation of the interior voxels of a cluster *k*:

22 $$\begin{aligned} \sigma _x^{(k)}=std\{\mu _x{(v)}:v\in V_{k^{int}}\},\quad \sigma _n^{(k)}=std\{\mu _n{(v)}:v\in V_{k^{int}}\}. \end{aligned}$$

The standard deviation have the same cm$^{-1}$ as the centroids, and represent the axial dispersion of the clusters.

##### Davies–Bouldin index

A scalar that calculates the spread of each material by the root-mean-squared (RMS) of its standard deviations:

23 $$\begin{aligned} s_k=\sqrt{\frac{(\sigma _x^{(k)})^2+(\sigma _n^{(k)})^2)}{2}}, \end{aligned}$$

and with $d(k,j)=||\hat{\mu }_k-\hat{\mu }_j||$ be the distance between two materials’ label-anchored centroids, the Davies-Bouldin index^31^ is calculated as follows:

24 $$\begin{aligned} DB=(1/M')\sum _{k=1}^{M'}max_{j\ne k} [(s_k+s_j)/d(k,j)]. \end{aligned}$$

The Davies–Bouldin index is dimensionless and cannot be lower than 0. *DB→ 0* implies perfectly separated, point-like clusters, while higher *DB ≫ 1* show overlap between clusters.

##### Pairwise material overlap

Interpreting the label-anchored centroids ${\hat{\mu }_k}$ as Voronoi seeds for tessellation, and for every unordered pair of materials (*a*, *b*) whose centroids are within a threshold (defined at $d_{max}=2$ cm$^{-1}$), every voxel *v* is classified with a ground-truth label $L_v \in {a,b}$ by nearest centroid:

25 $$\begin{aligned} \hat{y}_v=arg min_{k\in {a,b}} ||p_v-\hat{\mu }_k||^2, \end{aligned}$$

where $p_v=(\hat{\mu }_x^{v},\hat{\mu }_n^{v})$. The pairwise overlap fraction is calculated as:

26 $$\begin{aligned} O_{ab}=\#\{v:L_v\in \{a,b\}\wedge \hat{y}_v \ne L_v\} / \#\{v:L_v\in \{a,b\}\}, \end{aligned}$$

where $O_{ab}\in [0,1]$ represents the pairwise classification error rate between two materials.
