# Supplementary material for: Introducing DIANA: dual-mode imaging analysis open-source simulation software
Source: Sci Rep. 2026 Aug 11;16:24943. doi: 10.1038/s41598-026-65869-8 (PMC13462087; doi:10.1038/s41598-026-65869-8)
Supplement: Supplementary file 1 [file 41598_2026_65869_MOESM1_ESM.pdf]

Supplementary Information

The quality metrics of the iterative algorithms (SIRT, SART, CGLS) depend on the number of iterations. To quantify this dependence, the composite phantom of the main text was reconstructed under the same conditions as Table 1 (1024^3 voxels, 1200 projections, polychromatic 120 kVp X-ray spectrum, thermal neutrons, no injected artifacts) with SIRT and SART at 50, 100, 150 and 200 iterations and with CGLS at 10, 20, 30 and 50 iterations, and the full metric pipeline was applied to each reconstruction. The iteration counts used for the results reported in the main text (SIRT: 50, SART: 50, CGLS: 30) are marked in Supplementary Fig. S1 and Table S1.

Supplementary Figure S1 shows the mean centroid error CE, the Davies–Bouldin index DB, and the iron-cluster metrics ε_k(Fe) and E_k(Fe) as a function of the iteration number, with the (non-iterative) FBP result as a reference (slices of the reconstruction of the composite phantom using the FBP are shown in Fig. S2). From the graph, some data interpretation can be made: First, CGLS converges rapidly: by approximately 30 iterations it reaches the FBP-like least-squares solution (CE, DB and E_k(Fe) within $\approx 2\%$ of the FBP values), and the metrics change by less than 1% between 30 and 50 iterations; Second, SIRT and SART evolve monotonically toward the same solution but have not fully converged at 200 iterations; Third, the low-iteration regime of SIRT and SART is the over-smoothed regime: voxel values are drawn toward population-averaged (μ_x, μ_n) pairs, which lowers the mean centroid error while degrading cluster separability (high DB) and suppressing the streak morphology of the dense phases (compact E_k(Fe)). As the iterations increase, the metallic centroid error grows and the clusters elongate toward the FBP-like streaked solution, while the separability improves. The apparent CE advantage of SIRT and SART at their reported operating points is therefore a property of early stopping, not a more accurate reconstruction of the dense phases, which reinforces the conclusion that no single metric can faithfully rank reconstruction quality.

Table S1: Metric values and reconstruction times for the iteration sweep shown in Supplementary Fig. S1. FBP is non-iterative and serves as the reference. Daggers (†) mark the iteration counts used for the results of the main text.

**Algorithm**	**Iter.**	**CE** (cm^{-1})	**DB**	$\boldsymbol{\varepsilon_k}$**(Fe)** ($\text{cm}^{-1}$)	$\boldsymbol{\varepsilon_k}$**(Ti)** ($\text{cm}^{-1}$)	$\boldsymbol{E_k}$**(Fe)**	$\boldsymbol{E_k}$**(Ti)**	**Time (s)**
FBP	–	1.30	0.20	4.04	2.02	122.6	29.3	26
SIRT	50†	1.08	0.52	3.09	1.48	3.1	1.5	315
SIRT	100	1.19	0.39	3.62	1.78	4.2	1.5	606
SIRT	150	1.23	0.33	3.79	1.88	8.4	1.8	895
SIRT	200	1.25	0.29	3.87	1.93	12.8	2.4	1185
SART	50†	1.11	0.64	3.12	1.49	3.6	2.0	44
SART	100	1.21	0.48	3.64	1.79	4.9	2.0	65
SART	150	1.24	0.39	3.81	1.89	9.1	2.2	85
SART	200	1.26	0.34	3.90	1.94	13.9	2.8	105
CGLS	10	1.26	0.36	3.89	1.94	11.0	2.1	86
CGLS	20	1.30	0.23	4.05	2.03	50.2	8.6	148
CGLS	30†	1.30	0.21	4.06	2.03	95.3	16.2	211
CGLS	50	1.30	0.21	4.06	2.03	66.5	13.6	334

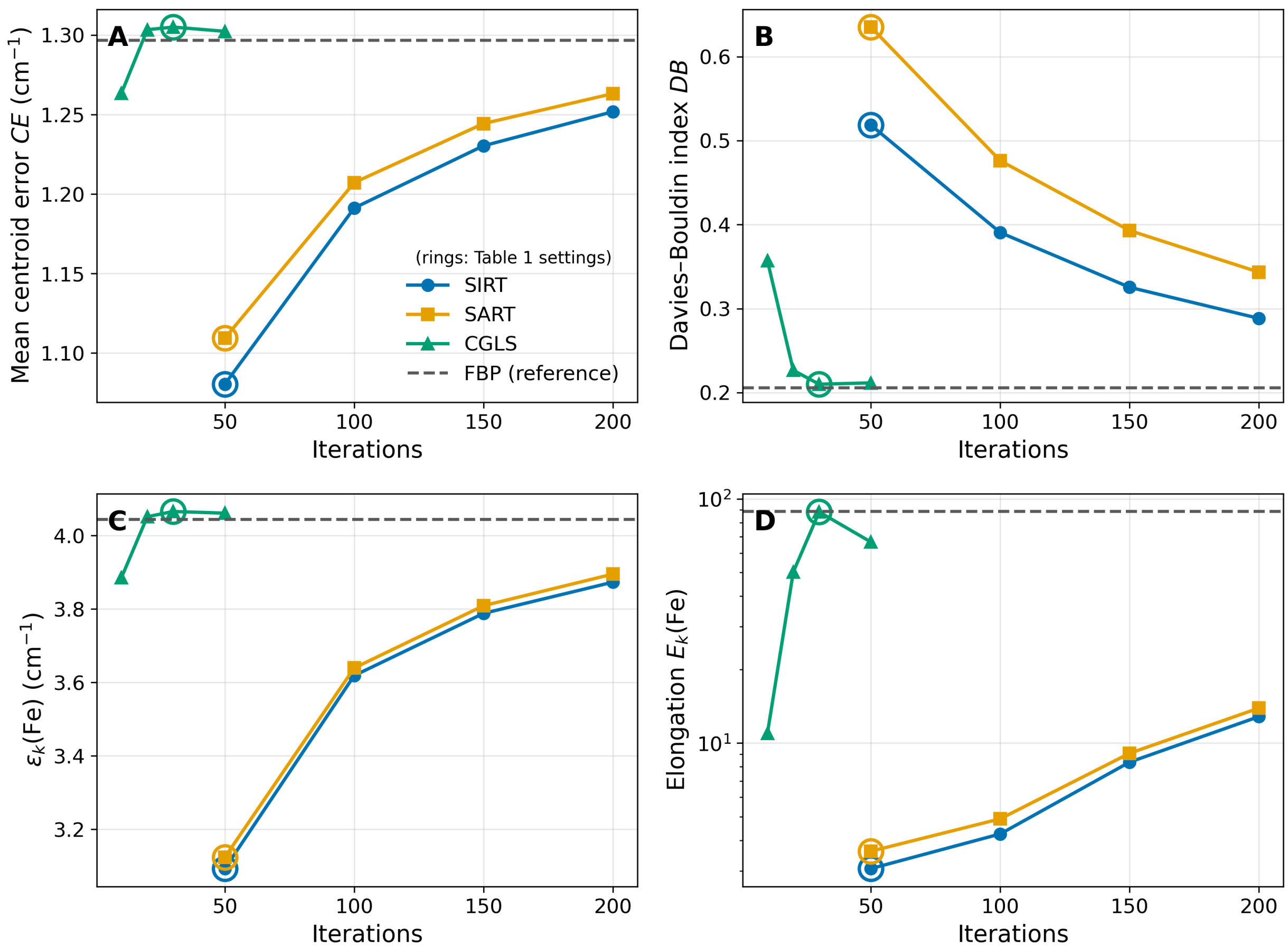

Figure S1: Iteration dependence of the quality metrics for the composite phantom, simulated under the conditions of Table 1 of the main text (1024^3 voxels, 1200 projections, 120 kVp polychromatic X-ray spectrum, thermal neutrons, no injected artifacts). **A**: mean centroid error CE; **B**: Davies–Bouldin index DB; **C**: per-material centroid error of iron ε_k(Fe); **D**: iron cluster elongation E_k(Fe) (logarithmic scale). Dashed grey lines mark the FBP reference; open rings mark the iteration counts used in the main text (SIRT: 50, SART: 50, CGLS: 30). SIRT and SART migrate monotonically from an over-smoothed, low-CE/high-DB regime toward the FBP-like solution, while CGLS reaches that solution within ≈30 iterations.

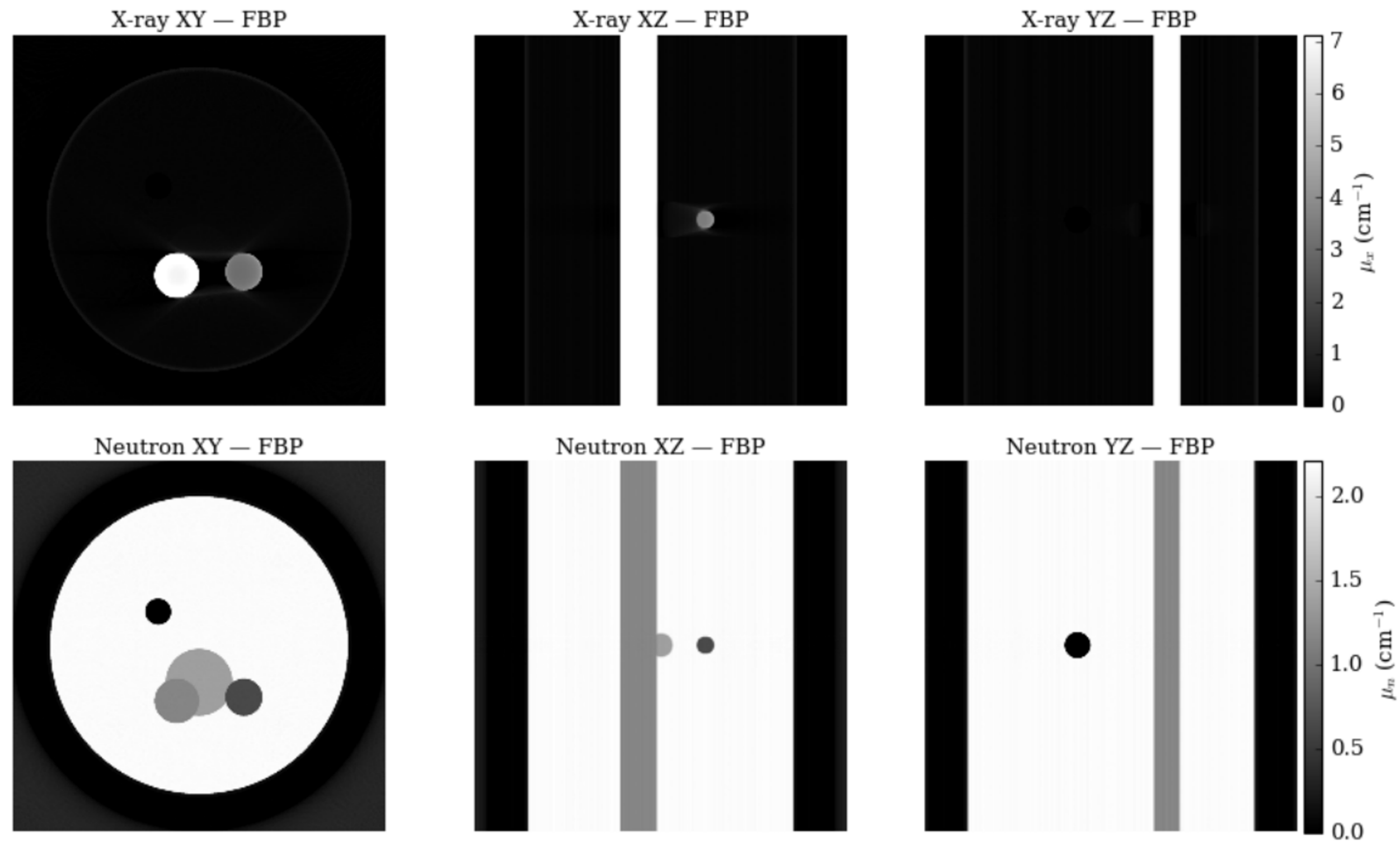

Figure S2: Slices showing sections of the reconstructed volumes, both X-rays and neutrons, of the composite phantom using the Filtered Back-Projection.

Table S2: Material parameters used to build the ground-truth phantoms. Neutron coefficients are separated into their absorption, coherent and incoherent scattering components, evaluated at 25 meV (thermal, $\lambda \approx 1.798$ Å); μ_n is their sum. X-ray values are quoted at 80 keV, the tabulated energy at which the ground-truth histogram coordinates are defined. Materials marked *XCOM / tab.* take their X-ray attenuation directly from a 13-point NIST XCOM table spanning 20–300 keV; those marked *formula* + ρ are computed from the stoichiometric formula and mass density by mass-fraction weighting of the elemental NIST XCOM coefficients, with neutron cross-sections taken from the standard thermal-neutron tables. All attenuation coefficients are in cm^{-1}.

**Material**	**Symbol**	$\boldsymbol{\rho}$ (g cm^{-3})	μ_n^{abs}	μ_n^{coh}	μ_n^{inc}	μ_n	μ_x	**Source**
Composite phantom								
Air	Air	0.0012	0	0	0	0	∼0	XCOM / tab.
Aluminum	Al	2.70	0.0140	0.0830	0.0010	0.0980	0.278	XCOM / tab.
HDPE	HDPE	0.95	0.0270	0.3690	1.7840	2.1800	0.167	XCOM / tab.
Water	H_2O	1.00	0.0220	0.2590	1.0990	1.3800	0.184	XCOM / tab.
Iron	Fe	7.87	0.2170	0.9100	0.0330	1.1600	4.120	XCOM / tab.
Titanium	Ti	4.51	0.3450	0.1750	0.1200	0.6400	1.480	XCOM / tab.
Lithium-ion cell phantom								
Steel (Fe–Ni)	Steel	7.85	0.2198	0.9523	0.0419	1.2140	4.695	formula + ρ
Copper	Cu	8.96	0.3130	0.7600	0.0470	1.1200	4.920	XCOM / tab.
NMC811	NMC811	4.80	2.3516	0.5887	0.1664	3.1067	2.335	formula + ρ
Graphite	Graphite	2.26	0.0004	0.6290	0.0001	0.6295	0.364	formula + ρ
PE separator + $LiPF_6$	Sep+Etl	1.05	0.1600	0.2206	1.1929	1.5735	0.186	formula + ρ
Aluminum	Al	2.70	0.0140	0.0830	0.0010	0.0980	0.278	XCOM / tab.
